# Reentrant superconductivity at an oxide heterointerface

**DOI:** 10.1126/sciadv.aeg0460

**Published:** 2026-06-24

**Authors:** Denis Maryenko, Minoru Kawamura, Igor V. Maznichenko, Sergey Ostanin, Ding Zhang, Markus Kriener, Vitalii K. Dugaev, Evgeny Ya. Sherman, Arthur Ernst, Masashi Kawasaki

**Affiliations:** ^1^ RIKEN Center for Emergent Matter Science (CEMS), Wako 351-0198, Japan.; ^2^ Institute of Physics, Martin Luther University Halle-Wittenberg, 06099 Halle (Saale), Germany.; ^3^ State Key Laboratory of Low Dimensional Quantum Physics and Department of Physics, Tsinghua University, Beijing, China.; ^4^ Department of Physics and Medical Engineering, Rzeszów University of Technology, 35-959 Rzeszów, Poland.; ^5^ Department of Physical Chemistry and EHU Quantum Center, University of the Basque Country UPV/EHU, Bilbao 48080, Spain.; ^6^ Ikerbasque, Basque Foundation for Science, Bilbao 48009, Spain.; ^7^ Institute for Theoretical Physics, Johannes Kepler University, 4040 Linz, Austria.; ^8^ Max Planck Institute of Microstructure Physics, D-06120 Halle, Germany.; ^9^ Donostia International Physics Center (DIPC), Manuel Lardizabal Ibilbidea, 4, 20018 Donostia/San Sebastián, Gipuzkoa, Spain.; ^10^ Department of Applied Physics and Quantum-Phase Electronics Center (QPEC), The University of Tokyo, Tokyo 113-8656, Japan.

## Abstract

A magnetic field typically suppresses superconductivity by either breaking Cooper pairs via the Zeeman effect or inducing vortex formation. However, under certain circumstances, a magnetic field can stabilize superconductivity instead. This seemingly counterintuitive phenomenon is associated with magnetic interactions and has been extensively studied in three-dimensional materials. By contrast, this phenomenon, hinting at unconventional superconductivity, remains largely unexplored in two-dimensional systems, with moiré-patterned graphene being the only known example. Here, we report the observation of reentrant superconductivity at the epitaxial (110)-oriented LaTiO_3_-KTaO_3_ interface. This phenomenon occurs across a wide range of charge carrier densities, which, unlike in three-dimensional materials, can be tuned in situ via electrostatic gating. We propose that the observed reentrant superconductivity can arise from an interplay between strong spin-orbit coupling and a magnetic field–driven modification of the Fermi surface. Our findings provide insight into reentrant superconductivity and establish a robust platform for exploring unconventional superconducting phenomena in two-dimensional systems.

## Teaser

At a two-dimensional oxide interface, superconductivity is suppressed by magnetic field and stabilizes at higher field.

## INTRODUCTION

The behavior of superconductors under magnetic fields offers profound insights into the properties of the superconducting phase and related emergent quantum states. Magnetic fields conventionally suppress superconductivity by breaking the coherence of Cooper pairs. However, in rare cases, they can stabilize the superconducting state (*1*–*4*). One notable example is the Jaccarino-Peter (JP) effect (*5*). Here, an applied external magnetic field compensates for an effective internal exchange field, which cancels the polarization of the conduction electrons. If the attractive interaction between electrons is sufficiently strong, then the superconducting phase can be stabilized in the compensation region. Originally proposed and later demonstrated in a number of ferromagnetic materials (*6*, *7*), the reentrant superconductivity (RSC) has been also observed in organic superconductors (*8*) and is discussed in the context of the heavy fermion system UTe_2_ (*9*). A different mechanism for RSC at high magnetic fields arises in heavy-fermion compounds (*3*, *4*). Here, the transition between low-field and high-field superconducting phases is accompanied by strong ferromagnetic fluctuations, which originate from the quantum instability of the ferromagnetic phase. The reentrant superconducting phase is considered unconventional with suggested spin-triplet pairing. Thus, the RSC arises from the intricate interplay between multiple degrees of freedom—particularly magnetism—and has long been a paradigm studied in three-dimensional (2D) materials.

By contrast, the occurrence of RSC in 2D systems remains largely unexplored. 2D systems provide a fundamentally distinct platform, where reduced dimensionality and broken inversion symmetry give rise to electronic states different from those in the bulk. In these systems, strong spin-orbit coupling (SOC) profoundly influences the superconducting phase. For instance, in the presence of a Rashba-type SOC, the superconducting order parameter can develop a mixed-parity state, combining both even- and odd-parity components (*10*). Despite growing interest in 2D superconducting materials, which have already revealed fascinating superconducting properties (*11*–*17*), the RSC has been observed so far only in magic angle twisted trilayer graphene (*18*). However, this system lacks strong SOC and pronounced magnetism, leaving the origin of the RSC in graphene uncertain.

Here, we report the observation of RSC at the (110)-oriented epitaxial interface of LaTiO_3_-KTaO_3_, a system fundamentally distinct from previously studied reentrant superconductors. Unlike other materials where RSC has been observed so far, the LaTiO_3_-KTaO_3_ interface does not provide a clear experimental evidence for electronic correlations or intrinsic magnetism. Instead, our system features a reduced dimensionality and strong SOC. KTaO_3_ is a cubic perovskite oxide with a conduction band derived from the 5*d* orbitals of the Ta atoms, known for their strong spin-orbit interaction. Its interface can exhibit a sizable Rashba- or/and Dresselhaus-type SOC, and its superconducting properties strongly depend on the crystal plane orientation (*19*–*21*). Recent studies, primarily on (111)-oriented interfaces, suggest the formation of unconventional superconducting phases, including evidence of a stripe phase (*19*), spontaneous symmetry breaking (*22*), and current direction–dependent resistive superconducting transitions, with different $T_{c}$ values extracted for transport along different in-plane crystallographic directions (*23*).

## RESULTS

### LaTiO_3_-KTaO_3_ heterostructure

**Fig. 1.**
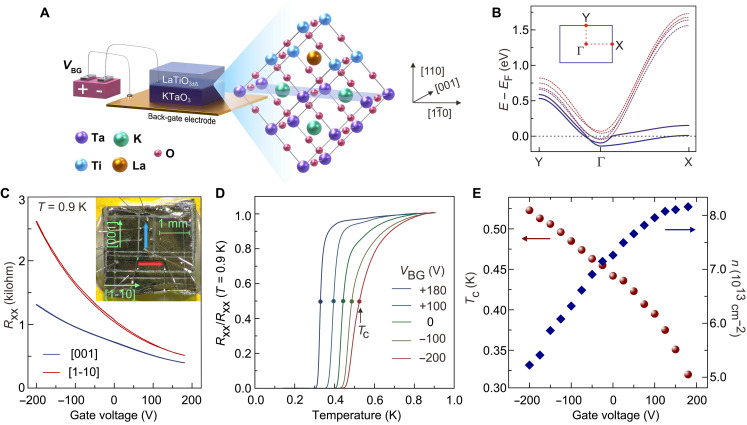
Properties of the (110)-oriented LaTiO_3_-KTaO_3_ interface. (**A**) Sketch of the interface between LaTiO_3_ and KTaO_3_. The backside of KTaO_3_ acts as an electrode to apply an electrostatic field to tune the in situ interface electronic properties. (**B**) Relativistic band structure of a 2D electron gas emerging at the (110) interface, which was simulated from first principles. Nearly flat-band regions around $E_{F}=0$ along Γ-X near the Brillouin zone boundary are formed almost equally by highly localized (l=2,m=−1) states of Ta and Ti. Solid lines are bands that are important for the theory model presented below. Dashed lines are other bands. The inset shows the unit cell in real space. (**C**) The back-gate voltage can effectively tune the sample resistance. Inset depicts the photograph of the device under study. The device allows to flow the current along the [001] and [1-10] crystallographic directions independently. (**D**) Superconducting transition at different back-gate voltages $V_{\text{BG}}$, measured with current applied along the [1-10] direction. The superconducting transition temperature $T_{c}$ is defined as a temperature at which the resistance has dropped by 50% from its value at normal conducting state. (**E**) Gate dependence of the superconducting transition temperature $T_{c}$ and the charge carrier density n, measured with current applied along the [1-10] direction.

Figure 1A schematically illustrates the (110)-oriented LaTiO_3_-KTaO_3_ interface, which is grown using a pulsed laser deposition technique (*24*). In this orientation, not only the interface breaks the inversion symmetry, but also the in-plane [001] and [$1\bar{1}0$] crystal directions are no longer equivalent. This asymmetry is reflected in the band dispersions plotted in Fig. 1B along Γ-X and Γ-Y. For the reciprocal direction Γ-Y, which is related to the lattice vector [$1\bar{1}0$], all bands crossing the Fermi energy $E_{F}$ have relatively high velocities. However, for Γ-X direction, the two lowest conduction bands become nearly flat near the Brillouin zone boundary. This is a typical feature of the band asymmetry computed within the (110) geometry that inevitably appears as a single flat Γ-X band in insulating KTaO_3_ (110) above its bandgap. In the case of the (110) LaTiO_3_-KTaO_3_ interface, the two flat bands, which are formed almost equally by the *d*-states of the Ta-Ti pairs, appear around $E_{F}$ along Γ-X. As a result, the Fermi surface of LaTiO_3_-KTaO_3_ (110) is elongated along this direction, which can change the transport properties measured along [110] and [100]. The effects of the SOC, magnetic exchange interactions, and spin noncollinearity, which were included in the ab initio calculations, determine the flat-band splitting and detailed Fermi surface, as well as its spin texture (fig. S1 and Supplementary Text).

The electronic characteristics of the interface are probed through electrical transport measurements. A photograph of the device under study is shown in the inset of Fig. 1C. The current can be injected along two orthogonal in-plane crystallographic directions using independently defined conductive channels. A back-gate voltage $V_{\text{BG}}$, applied between the backside of the KTaO_3_ substrate and the interface, tunes the channel conductance. Within the applied gate voltage range of −200 to +180 V, no measurable gate leakage current is detected. Furthermore, the hysteresis for $R_{\text{xx}}$ in gate sweeps is barely visible in Fig. 1C, ensuring a reliable device operation as a field-effect transistor. For all applied gate voltages, the interface undergoes a superconducting transition. Figure 1D exemplifies the temperature dependence of $R_{\text{xx}}$ near the superconducting transition for selected $V_{\text{BG}}$ values. For clarity of the presentation, $R_{\text{xx}}$ is normalized to its value at 0.9 K, where the superconductivity is suppressed. Figure 1E shows that the superconducting transition temperature $T_{c}$ increases as $V_{\text{BG}}$ decreases, while the charge carrier density, estimated from the Hall effect at 0.9 K, decreases accordingly. The gate tunability of $T_{c}$ follows a similar trend to that observed in (111)-oriented KTaO_3_ interfaces (*20*). However, unlike the (111) case, we also observe a substantial tunability of the charge carrier density with $V_{\text{BG}}$.

### Reentrant superconductivity

**Fig. 2.**
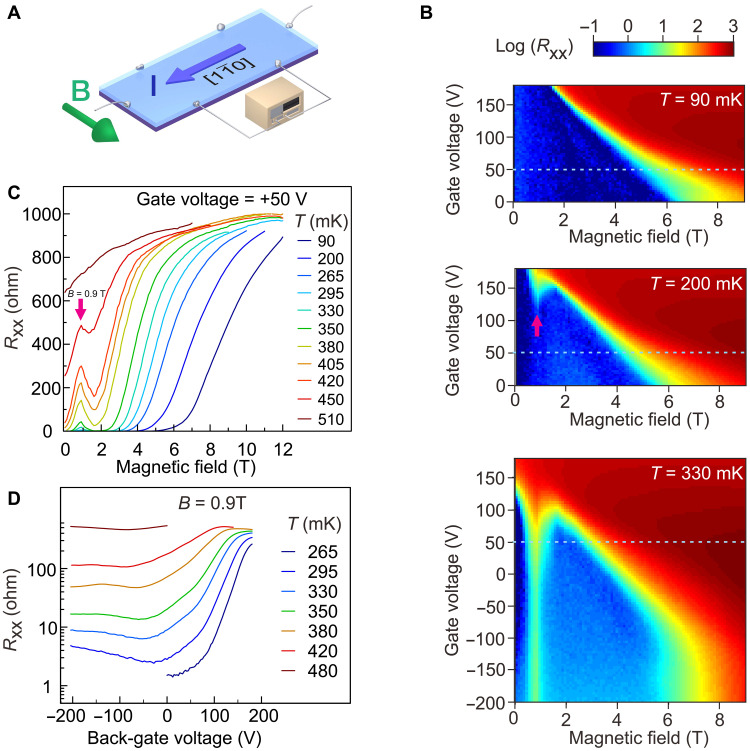
Experimental visualization of reentrant superconducting response. (**A**) Scheme of the experimental arrangement. (**B**) Color rendition plot of the longitudinal resistance $R_{\text{xx}}$ as functions of the gate voltage and the magnetic field. The reentrant feature becomes more pronounced with increasing temperature. (**C**) Magnetoresistance $R_{\text{xx}}$ traces for various temperatures at $V_{\text{BG}}=+50$ V. A resistive peak emerges at B=0.9 T as the temperature increases. (**D**) Gate voltage dependence of the amplitude of the resistive peak at several temperatures.

The central result of this work is summarized in Fig. 2, where the magnetic field is applied in-plane as sketched in Fig. 2A. The longitudinal resistance $R_{\text{xx}}$ is measured at various temperatures upon sweeping the back-gate voltage at discrete magnetic field values. This approach eliminates temperature variations that could arise from sweeping the field. Figure 2B presents a color rendition of $R_{\text{xx}}$ on a logarithmic scale at 90 mK, plotted as functions of the magnetic field and the gate voltage. The critical field required to suppress superconductivity increases as $V_{\text{BG}}$ decreases, consistent with the dependence of $T_{c}$ on the gate voltage (Fig. 1E). Notably, at this low temperature, the critical field already exceeds the laboratory magnetic field of 9 T, despite the limited gate range of 0 to +180 V. As expected, increasing the temperature reduces the critical field at which superconductivity is destroyed. However, beyond this anticipated trend, Fig. 2B reveals the emergence of a resistance cusp at B=0.9 T, as indicated by the red arrow. This feature becomes more pronounced with increasing temperature, as shown in Fig. 2B at T=330 mK. The cusp appears consistently at B=0.9 T, independent of both temperature and gate voltage. Thus, upon sweeping the magnetic field, the superconducting transport response is first suppressed (indicated by $R_{\text{xx}}\neq 0$) and then reappears at higher field, giving rise to a nonmonotonic, reentrant transport signature. We refer to this behavior as a reentrant superconducting response. Similar behavior is also observed when the current flows along [001] direction (fig. S2). To highlight this behavior, Fig. 2C shows $R_{\text{xx}}$ traces at $V_{\text{BG}}=+50$ V as a function of magnetic field for various temperatures. At low temperatures, the 0.9 T resistance peak is not observable, but as the temperature increases, it gradually emerges. We have qualitatively reproduced this result in another independently grown LaTiO_3_-KTaO_3_ (110)-oriented heterostructure, confirming the robustness of our observation (fig. S4).

One of the primary mechanisms that can suppress superconductivity is Zeeman splitting, which breaks Cooper pairs by aligning electron spins. In conventional Bardeen-Cooper-Schrieffer superconductors, assuming a g-factor of g=2, the critical field is given by the Clogston-Chandrasekhar paramagnetic limit $B_{P}=1.86\times T_{c},$ where $T_{c}$ is the superconducting temperature in Kelvin, and $B_{P}$ is given in Tesla (*25*, *26*). In our system, this places $B_{P}$ in the range of 0.6 to 0.9 T, depending on the gate voltage. The resistive peak at 0.9 T appears close to this paramagnetic limit. However, unlike $T_{c}$, the peak position is independent of the gate voltage, and its amplitude exhibits a nonmonotonic dependence on $V_{\text{BG}}$. As shown in Fig. 2D, the peak amplitude reaches a minimum around $V_{\text{BG}}=-50$ V and increases again for more negative $V_{\text{BG}}$. This suggests that the peak is unlikely to be directly related to the Zeeman effect. Another relevant field scale is set by the orbital coupling due to the applied in-plane magnetic field. As an order-of-magnitude geometric estimate for a simple orbital effect of an in-plane field, we take the relevant field scale to satisfy $B_{∥}\xi w≅\phi_{0}$, where $\phi_{0}$ is the magnetic flux quantum and ξ is the coherence length extracted from perpendicular field measurements (fig. S3). Using ξ extracted from perpendicular field measurements, the field scale of 0.9 T implies w≈45 nm, which far exceeds the typical conducting layer thickness at oxide interfaces, estimated to be around or even less than 10 nm. This disfavors a simple in-plane orbital origin of the resistance peak.

As discussed above, field-enhanced or field-restored superconductivity is sometimes associated with the JP effect, where an applied field compensates an internal exchange field generated by magnetic moments. In our structures, however, we find no evidence either for intrinsic magnetic order or for the ordering produced by external field: Neither the Hall signal nor the longitudinal resistance shows hysteresis, and the Hall response remains linear in field. Our relativistic first-principles calculations for LaTiO_3_/KTaO_3_ (110) indicate that moments induced at interfacial Ta are extremely small ≤0.003 μ*_B_*. Although defect-related paramagnetic moments (e.g., from oxygen vacancies or interfacial disorder) cannot be ruled out (*27*, *28*), we examine below whether a JP-type compensation scenario can plausibly account for our observations. A JP scenario requires an antiferromagnetic exchange coupling to oppose the applied Zeeman field. If magnetic moments are present, their microscopic origin (defects, interfacial disorder, or magnetic proximity) is itself uncertain, and both the sign and effective strength of the resulting exchange coupling can depend on that origin. Accordingly, the JP-required coupling is not known a priori for KTaO_3_-based interfaces. We therefore proceed by estimating the exchange-field scale that a JP scenario would require at the observed reentrant field. The characteristic field scale for the reentrant feature is $B^{*}\approx 0.9$ T, corresponding to the Zeeman scale $\mu_{B}B^{*}\approx$ 40 to 50 μeV. When the spin system is fully polarized, the effective magnetization can be estimated (*5*) as $H_{\text{eff}}∼{\mathrm{Jn}}_{m}/\mu_{B},$ where J is the exchange integral and $n_{m}$ is the concentration of magnetic impurities per unit cell. To have a substantial influence on the superconductivity, $H_{\text{eff}}$ should exceed the actual pair-breaking field B, being close to 6 T and greatly exceeding the Clogston-Chandrasekar limit due to the strong SOC. Assuming a relatively large $n_{m}∼10^{-3}$, this requires J∼0.3 eV, which is far larger than expected for dilute, weakly hybridized defects.

To further clarify the constraints on the applicability of the JP mechanism in our system, we proceed with the observation that the characteristic field of the reentrant feature is essentially temperature independent. Since the system we consider is not intrinsically magnetic, it is reasonable to consider that its B-induced magnetization is produced by diluted paramagnetic moments. Thus, in this case, the field scale associated with the onset of the exchange compensation in the JP model is expected to follow the moment polarization M∝tanh(x), with $x=\text{μB}/\left(k_{B}T\right)$. For an illustrative moment $\mu =\mu_{B}$, being a conservative upper bound for dilute defects, increasing T from 0.3 to 0.5 K leads to a substantial reduction of magnetic polarization at relevant $B^{*}\approx 0.9$ T. A characteristic compensation field scale would therefore be expected to shift to higher B as temperature increases. Experimentally, however, the peak remains at $B^{*}\approx 0.9$ T. The absence of any gate dependence of $B^{*}$, despite a pronounced gate dependence of $T_{c}$ (Fig. 1E), further suggests that this characteristic field scale is not primarily set by the superconducting energy scale.

Moreover, the reentering feature is confined to a relatively narrow field range ΔB≈0.3 around $B^{*}.$ At fixed T, this corresponds to only a modest change in the polarization parameter $x=\mu B/\left(k_{B}T\right)$ (of the order of 30%). For x≳1, the moments are already close to saturation, so the exchange field varies only weakly across this range. Conversely, for x≲1, the moments remain weakly polarized, so producing a sizable exchange compensation over such a narrow ΔB would require an unusually large effective coupling. In either regime, generating a pronounced reentrant feature within a narrow field interval is not straightforward in a simple JP picture.

Together, these considerations do not rule out a JP contribution, but they constrain the magnetic interaction it would require—sizable localized moments together with sufficiently strong antiferromagnetic exchange capable of producing compensation on the scale $B^{*}$. Given the lack of evidence that such conditions are realized in our structures, we focus instead on mechanisms arising from the interfacial electronic structure under an in-plane magnetic field, where strong SOC is expected to play a central role.

## DISCUSSION

A notable feature of our system is its high parallel critical field, which exceeds the perpendicular critical field by more than an order of magnitude. Such a large anisotropy is commonly associated with strong SOC, which can substantially enhance the pair-breaking field, as demonstrated, for example, in transition metal dichalcogenides (*11*–*13*). In those materials, Ising-type SOC locks electron spins out-of-plane and prevents their alignment with an external magnetic field. By contrast, at the LaTiO_3_-KTaO_3_ interface, Rashba- and Dresselhaus-type couplings are expected, leading to an in-plane spin texture and allowing a mixed singlet-triplet superconducting state already at zero field (*10*). An applied magnetic field can modify the relative weight of singlet and triplet components. However, this spin mixing by itself does not explain the RSC phenomenon. We therefore turn to additional consequences of interfacial SOC in an in-plane field, which form the basis of the minimal model introduced below.

The effect of RSC can be qualitatively viewed as a nonmonotonic dependence of the superconducting transition temperature $T_{c}\left(B\right)$ on the magnetic field B. Specifically, if $T_{c}\left(B\right)$ dependence has a minimum, $T_{c}^{\text{min}}$ at a certain field $B_{\text{min}}$, then at an experimental temperature $T>T_{c}^{\text{min}}$, a finite resistive interval appears between two field ranges exhibiting superconducting transport. At low temperatures, $T<T_{c}^{\text{min}}$, this resistive interval does not appear.

We show that such a minimum can arise from the combined effect of (i) the van Hove singularity near the Brillouin zone boundary seen in our ab initio calculations (Fig. 1B) and (ii) interfacial SOC, which has a strong contribution from heavy Ta atoms. Structural inversion asymmetry at the interface naturally generates Rashba-type SOC. However, a more detailed symmetry analysis shows that oxide interfaces can also host Dresselhaus-like terms, originating from asymmetry of the interatomic bonds at the interface (*29*–*31*). Near the zone boundary, the effective SOC can therefore contain both Rashba- and Dresselhaus-like contributions, which generate different momentum-dependent spin orientations and coupling to an in-plane magnetic field.

**Fig. 3.**
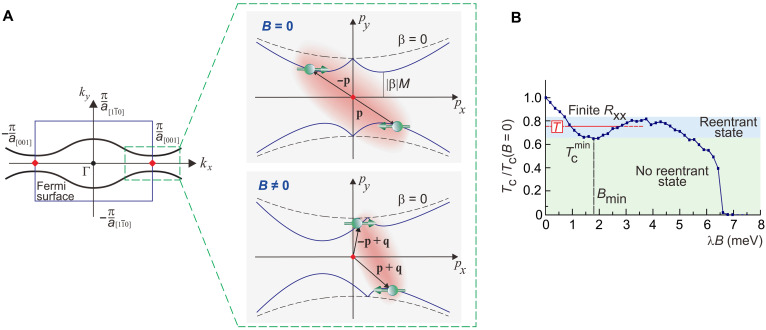
Theoretical model to explain the observed reentrant transport response. (**A**) Left: Brillouin zone and Fermi surface demonstrating the van Hove singularity in the absence of SOC at the Brillouin zone boundary. The lattice constants are $a_{\left[1\bar{1}0\right]}$ and $a_{\left[100\right]},$ respectively. Right: zoom in on the states near the van Hove singularity taking into account SOC and magnetic field. Dashed lines correspond to the Fermi surface without SOC. Solid lines, marked with the corresponding spin orientations, show the deformation of the Fermi surface by SOC and magnetic field. The Fermi surface states contributing to the Cooper pairing are shown as filled circles. Without loss of generality, in this figure, we assume $\beta =\beta_{x}<0$. (**B**) Numerical result demonstrating the existence of the required for the RSC minimum in the $T_{c}\left(B\right)$ dependence. $T_{c}$ is normalized to its value at B=0.

According to the ab initio results and the above presented symmetry arguments, we consider electrons near the extended saddle point (see Fig. 3A) with the effective mass Hamiltonian (we use ℏ≡1 units) and anisotropic SOC as

$$H=-\frac{p_{x}^{2}}{2M}+\frac{p_{y}^{2}}{2m}+\left(\beta_{x}p_{x}-\alpha_{y}p_{y}+\lambda B\right)\sigma_{x}-\left(\beta_{y}p_{y}-\alpha_{x}p_{x}\right)\sigma_{y}$$ (1)

Here, $p_{x}$ and $p_{y}$ are defined relative to the Brillouin zone boundary $\pi /a_{\left[001\right]}$ point (Fig. 3A), i.e., $p_{x}=k_{x}-\pi /a_{\left[001\right]},$ $p_{y}=k_{y},$ and M≫m. The constants $\left(\beta_{x},\beta_{y}\right)$ and $\left(\alpha_{x},\alpha_{y}\right)$ describe anisotropic Dresselhaus and Rashba couplings, respectively, and λ is the effective system-dependent Landé factor. The symmetry analysis indicates that near the singularity, the main effect related to the RSC in the magnetic field parallel to the x axis is due to the $\beta_{x}$-related coupling leading to the Dresselhaus-like contribution of the form $\sigma_{x}k_{x}.$ Thus, to demonstrate qualitatively the origin of the RSC, we therefore focus on the $\beta_{x}$-originated coupling.

At a given SOC, momentum-dependent spin splitting of electron band states produces a B-dependent deformation of the Fermi surface; the effect being most pronounced near the van Hove singularity. Fixing the chemical potential μ, we describe the Fermi surface of the upper spin split branch by

$$\frac{p_{y}^{2}}{2m}=\mu +\frac{p_{x}^{2}}{2M}-\left|\beta_{x}p_{x}+\lambda B\right|$$ (2)

At B=0, the momentum-dependent spin expectation value is set by SOC, $\langle \sigma_{x}\rangle =\text{sgn}\left(\beta_{x}p_{x}\right)$, and the Fermi contour has two symmetric extrema at $p_{x}=\pm \beta_{x}M$ (Fig. 3A). These extrema are well-displaced from the Brillouin zone boundary due to the large mass M. The corresponding Cooper pairing between p and −p is illustrated in Fig. 3A. A nonzero field B breaks the p↔−p symmetry through the shift $|\beta_{x}p_{x}|\to |\beta_{x}p_{x}+\lambda B|.$ In this model, the extrema of the upper branch remain at $p_{x}=\pm \beta_{x}M$, but the values of $p_{y}^{2}/2m$ at these points shift to $\mu -M\beta_{x}^{2}/2\pm \lambda B$, and the spin polarization follows the deformation of the Fermi surface and becomes $\langle \sigma_{x}\rangle =\text{sgn}\left(\beta_{x}p_{x}+\lambda B\right)$ (Fig. 3A). For sufficiently strong fields, $\lambda B>M\beta_{x}^{2}$, one extremum disappears. As a result, the field pushes one side of the Fermi contour closer to the saddle point region, enhancing the density of states while simultaneously generating a pronounced p↔−p asymmetry. This asymmetry favors pairing with a finite center-of-mass momentum (schematically shown in Fig. 3B), which reduces the pairing efficiency and tends to suppress $T_{c}$. At the same time, the enhanced density of states tends to increase $T_{c}$. The competition between these two effects produces a nonmonotonic $T_{c}\left(B\right)$ with a minimum (Fig. 3B and Supplementary Text). Consequently, for experimental temperatures $T<T_{c}^{\text{min}}$, the system remains superconducting up to a single critical field. This behavior is consistent with the experimental trends.

We notice that this approach is based on the field-dependent deformation of the Fermi surface that can occur near a van Hove singularity in the presence of the SOC. Additional interface-specific effects may further modify $T_{c}\left(B\right)$. We mention here two possibilities. The momentum-dependent pairing can lead to a modification of the effective pairing interaction and, thus, to a decrease in the transition temperature. In addition, if the superconductivity is mediated by ferromagnetic or antiferromagnetic fluctuations, which are possibly enhanced by a high density of states near the van Hove singularity, then a magnetic field can suppress the fluctuations and reduce $T_{c}$, while the field-induced increase in the density of states may restore $T_{c}$ at higher fields, producing reentrance.

Although our minimal model captures key qualitative features of the nonmonotonic in-plane field response, it is not intended to exclude a multiorbital (multiband) superconducting state at the LaTiO_3_/KTaO_3_ interface. If several Fermi pockets are occupied, then additional pairing channels become available, including both intra- and interband Cooper pairings. Theory indicates that an in-plane magnetic field can shift the balance between these channels, which can, in principle, yield separated superconducting regions and even an intermediate gapless regime (*32*–*34*). In multiorbital systems, finite-momentum interband pairing can also become favorable at higher fields. While we do not attempt a quantitative multiband description here, these considerations provide a plausible complementary framework for interpreting the field-driven suppression and recovery of superconductivity observed at this interface.

In summary, we report a robust reentrant superconducting response at the LaTiO_3_-KTaO_3_ interface under an in-plane magnetic field. The reentrant response persists over a wide gate voltage range, allowing it to be tracked as the carrier density is tuned electrostatically. To provide a qualitative understanding of the observed nonmonotonic field response, we introduce a minimal description based on two ingredients that are well motivated for this interface—an anisotropic electronic structure and strong interfacial SOC. The model reproduces the main qualitative trends and illustrates how reentrant behavior can arise from the interplay of SOC, band anisotropy, and an in-plane magnetic field, without magnetic compensation playing a dominant role. More generally, reentrant behavior has been discussed in terms of different microscopic mechanisms across material systems, with potential contributions from magnetism, multiband structure, and SOC. In LaTiO_3_-KTaO_3_, strong interfacial SOC is present, and the possible presence of defect- or proximity-related magnetic moments cannot be excluded. Our data do not support a JP-dominated compensation scenario and place constraints on the regime in which a JP mechanism could apply; a subdominant magnetic contribution cannot be excluded. Together, these features make the system well suited for future work aimed at disentangling electronic structure and magnetic contributions to field-driven changes of superconductivity in two dimensions.

## MATERIALS AND METHODS

### Thin-film growth

LaTiO_3_/KTaO_3_ (110) heterostructures were synthesized by pulsed-laser deposition using a polycrystalline La_2_Ti_2_O_7_ target (2 Hz, 1.6 J cm^−2^) on ~3 mm–by–3 mm KTaO_3_ (110) substrates. Growth was monitored in situ by reflection high-energy electron diffraction. The substrate was heated to 400°C, coated with a thin amorphous layer to protect the KTaO_3_ surface during further heating, and then heated to 700°C to induce solid-state epitaxial crystallization. The main LaTiO_3_ layer was subsequently deposited at 600°C, with thickness set by the pulse number. After cooling to room temperature and thermalization, the films were capped with a thin amorphous layer.

### Magnetotransport measurements

Magnetotransport measurements were performed in a dilution refrigerator equipped with a one-axis sample rotator, allowing alignment of the magnetic field with respect to the interface plane. Samples were electrically contacted by direct Al wire bonding and fixed with silver epoxy onto a metallized chip carrier, which also enabled application of a back-gate voltage. Transport was measured in a four-terminal configuration using a standard low-frequency lock-in technique with an excitation current of 100 nA at 13.3 Hz. The excitation current is well below the superconducting critical current. No measurable gate leakage current was detected for the applied back-gate voltages.

## Supplementary Materials

### This PDF file includes:

Supplementary Text Figs. S1 to S10 References
