# Supplementary material for: Reentrant superconductivity at an oxide heterointerface
Source: Sci Adv. 2026 Jun 24;12(26):eaeg0460. doi: 10.1126/sciadv.aeg0460 (PMC13292944; doi:10.1126/sciadv.aeg0460)
Supplement: Supplementary file 1 — Supplementary Text Figs. S1 to S10 References [file sciadv.aeg0460_sm.pdf]

Science Advances
AAAS

Supplementary Materials for

Reentrant superconductivity at an oxide heterointerface

Denis Maryenko *et al.*

Corresponding author: Denis Maryenko, maryenko@riken.jp

Sci. Adv. **12**, eaeg0460 (2026)
DOI: 10.1126/sciadv.aeg0460

This PDF file includes:

Supplementary Text
Figs. S1 to S10
References

Ab initio calculations

The density-functional theory calculations of the $LaTiO_3/KTaO_3$ (LTiO/KTO) heterostructure were performed using the code VASP (*35*). The structural optimization and electronic properties of LTiO/KTO were computed within the Perdew-Burke-Ernzerhof generalized gradient approximation (*36*) to the exchange-correlation potential. The electron-ion interactions were described by projector-augmented wave pseudopotentials. The electronic wave functions were represented by plane waves with a cutoff energy of 450 eV. Ionic relaxations were performed using the conjugate-gradient algorithm until the Hellmann-Feynman forces became less than 10^{-2} eV/Å. The (110) interfaces were simulated within the model of the superlattice, $(LTiO)_n/(KTO)_m$ (110), in which the number of the (110) oriented KTO and LTiO layers varied up to $n = 8$ and $m = 8$. After an accurate structural relaxation, the density of states (DOS) and the band structure of each $(LTiO)_n/(KTO)_m$ (110) were computed in the presence of spin-orbit coupling (SOC) using the noncollinear VASP option (*37*) and tetrahedron method on the Γ-centered and dense **k**-mesh. All other computational details regarding the Hubbard parameterization for strongly correlated Ti $3d$ states, its orbital ordering, and the TiO_6 octahedra tilting angles, can be found in Refs. (*38, 39*).

A simplified picture of the 2D electron gas (2DEG) emerging at the interface was simulated using the superlattice $(LTiO)_1/(KTO)_1$ (110) reduced down to $n = m = 1$. The corresponding relativistic band structure, $E(k_x, k_y, 0)$, which was calculated within the Brillouin zone plane $k = 0$ and which is plotted in Figure 1b (main text) along the two perpendicular directions $\Gamma - X$ and $\Gamma - Y$ near the Fermi level (E_F), clearly shows (i) the interface metallization and (ii) dramatically different dispersions that is in a good agreement with the measured anisotropic in-plane conductivity of LTiO/KTO (110). It should be noted that the typical mechanism of 2DEG, which is based on the polar layer-alteration discontunity at the oxide interfaces, is not applicable to the case of LTiO/KTO (110) where each material layer is not polar. 2DEG appears there due to a combination of the interfacial band bending and constrainedly undertilted TiO_6 from the LTiO side (*38*). For $(LTiO)_1/(KTO)_1$ (110), as Figure 1b (main text) shows, a few low conduction bands appear below E_F near the Brillouin zone centre Γ. For $\Gamma - Y$, all these bands, in which the Ta $5d$ states dominate over Ti $3d$ states, cross E_F with relatively high electron velocities $v(\mathbf{k})$, whereas the two lowest bands along $\Gamma - X$ seem very flat near the Brillouin zone boundary X and, thus, these bands can

form an open Fermi surface with very small Fermi velocities $v_F(\mathbf{k})$.

In Figure S1, the ($k_z = 0$) cross section of the calculated Fermi surface (FS) of $(LTiO)_1/(KTO)_1$ (110) shows that the presence of one or two open FS features along $\Gamma - X$ is quite possible. Regarding this open FS, its shape and topology can be tuned by applied external electric or/and magnetic fields. Below we model this scenario using the tight-binding model in the context of re-entrant superconductivity in LTiO/KTO (110). Our *ab initio* calculations demonstrate also that with increasing the thickness of LTiO/KTO (110) its FS becomes extremely complicated. However, the anisotropic flat bands and related FS features capable of being opened/closed is the key and remanent factor of the (110)-oriented interface.

The accurate first-principles calculations of magnetic configurations in $KTaO_3/LaTiO_3$ (110) were performed for the 4-u.c.-thick and 8-u.c.-thick overlayers of $LaTiO_3$ using the 20-Å-thick vacuum and also the superlattices with no vacuum. For all cases, the computed magnetic moments induced on interfacial Ta never exceed 0.003–0.004 μ_B. Regarding the $LaTiO_3$ side, each (110) layer which contains Ti is antiferromagnetic itself, while the Ti magnetic moments significantly decrease near the interface from the bulk value of 1 μ_B to about 0.2–0.3 μ_B depending on the model and overlayer thickness. The magnetic degradation of the LTiO interface can be explained by the TiO_6 undertilting (*38*). In our simulation, there are two interfacial Ti and after relaxation these antiferromagnetically ordered sites do not completely compensate each other showing the marginal disbalance of less than 0.1 μ_B.

To inspect the effect of oxygen vacancies we relaxed an isolated oxygen vacancy (V_O) in the 240-atom supercells of $KTaO_3$ and $LaTiO_3$ using the relativistic non-collinear option of VASP. For $KTaO_3$ the V_O formation energy of 5.4 eV is less by ~0.3 eV compared to that of $LaTiO_3$. It seems that oxygen vacancies in KTO/LTiO are not necessarily to be traped at the interface. In $KTaO_3$, oxygen vacancy induces negligible magnetic moment of $< 0.004\mu_B$ on the nearest Ta cations that differs the KTO substrate from widely used $SrTiO_3$ substrate, in which V_O may induce the magnetic moment on Ti. As for $LaTiO_3$, the simulated presence of isolated V_O does not destroy completely the antiferromagnetic G-type ordering of Ti. We found the decrease of Ti magnetic moment followed by uncompensated magnetisation of $< 0.1\mu_B$ near the vacancy position.

Thus, we can conclude that the KTO/LTiO (110) interface is marginally magnetic even at the presence of oxygen vacancies that explains the absence of the anomalous Hall effect there.

Re-entrant response for current along [001]

We also measured the magnetotransport response with current applied along the [001] crystal direction. The corresponding data are shown in Fig. S2. At the lowest temperature (T = 90 mK), no clear low-field resistive peak is resolved. As the temperature is increased, however, a resistive peak emerges in the same magnetic-field range as for transport along [1-10]. This shows that re-entrant-like behavior is also observed for current along [001], indicating that the effect is not limited to a single in-plane transport direction.

Measurements in perpendicular field

We measured the transport characteristics of the structure when the magnetic field is applied perpendicular to the interface as shown in Fig. S3a. We swept the gate voltage and the field to acquire color plots presented in Fig. S3b. The measurements are performed at several temperatures. Only the measurements at 50 mK and at 380 mK are shown here. Consistent with the T_c dependence on the gate voltage, the critical magnetic field is higher for a lower gate voltage. From the dependence:

$$B_C^{\perp} = \frac{\phi_0}{2\pi\xi_{[001]}\xi_{[1-10]}}\left(1 - \frac{T}{T_c}\right), \tag{S1}$$

where ϕ_0 is the flux quantum, we estimate the in-plane coherence length $\xi_{\text{eff}} = \sqrt{\xi_{[001]}\xi_{[1-10]}}$ for every gate voltage. Because of the in-plane anisotropy, this quantity should be regarded as the geometric mean of the coherence lengths along the two principal in-plane directions and is used here as an order-of-magnitude estimate Fig. S3c displays the ξ_{eff} dependence on the gate voltage.

Reproducibility of the re-entrant superconductivity

We have independently grown another $LaTiO_3$-$KTaO_3$ (110)-oriented structure. Figure S4 shows the color rendition plot of the longitudinal resistance R_{xx} as functions of the gate voltage and the magnetic field. The results of measurements at several temperatures are presented. They show that the re-entrant superconducting state is qualitatively reproduced. At low temperatures, there is no signature of the re-entrant behavior. As the temperature increases, a resistive peak develops at low field. Its position on the $B-$axis is independent of the gate voltage. This is in correspondence with the results presented in the main text.

Current-voltage characteristics

To further characterize the superconducting transport, we measured current-voltage (I–V) characteristics of the $LaTiO_3/KTaO_3(110)$ interface at zero magnetic field for several back-gate voltages at $T = 90$ mK. A representative I–V trace measured at $V_{BG} = 0$ V is shown in Fig. S5A. At low bias, the voltage remains near zero, consistent with superconducting transport, while a finite voltage develops above a threshold current. We define the critical current, I_c, as indicated in Fig. S5A from the onset of the voltage response.

The extracted I_c values are summarized in Fig. S5B as a function of back-gate voltage. The critical current is on the order of 20 μA and shows a clear gate-voltage dependence, confirming that the superconducting state is tunable by electrostatic gating. For comparison, all magnetotransport measurements presented in the main manuscript were performed with an excitation current of 100 nA, which is far below the measured critical current.

Out-of-plane magnetoresistance above T_c

For completeness, we measured the out-of-plane magnetoresistance above the superconducting transition temperature for several back-gate voltages. Figure S6 shows the normalized resistance, $R_{xx}/R_{xx}(B = 0)$, measured at $V_{BG} = -200$, 0, and 100 V. Within the resolution of the present measurements, no hysteretic behavior is observed.

Theory of re-entrant superconductivity

Model

We assume that electrons at the $LaTiO_3$-$KTaO_3$ (LTO-KTO) interface can be described by a two-dimensional model corresponding to overlapping atomic orbitals located at the Ta and Ti atoms in close proximity to the interface. In this tight-binding approximation with overlapping orbitals in the x- and y- directions, the energy corresponding to the wavevector $\mathbf{k} = (k_x, k_y)$ has the form

$$\varepsilon_{\mathbf{k}}^{(0)} = -w \cos(k_x a) - t \cos(k_y a), \tag{S2}$$

where a is the lattice constant. Although the crystal structure at the interface is anisotropic, we describe it with a single lattice constant attributing all anisotropy effects to the difference between t

and w. In the following, we assume $w \ll t$, which is related to different overlaps of wavefunctions residing at the Ti and Ta ions in the x and y directions and corresponding to the highly anisotropic effective mass. The transition to an open Fermi surface occurs at $\mathbf{k} = (\pm\pi, 0)$, where the $\varepsilon_{\mathbf{k}}^{(0)} = w - t$, with a saddle-like energy profile, according to the results of *ab initio* calculations.

We assume that the main effect of spin-orbit coupling on the states near the van Hove singularity at the Brillouin zone boundary is due to the Dresselhaus spin-orbit interaction (DSOI), which can be attributed to, e.g., the asymmetry of the interatomic bonds at the interfaces *(30-32)*. In the tight-binding approximation, the isotropic DSOI has the form

$$H_{\mathrm{so}} = \frac{\beta}{a}\left[\sigma_x \sin(k_x a) - \sigma_y \sin(k_y a)\right], \tag{S3}$$

with β being the coupling constant. For $k_x, k_y \ll 1/a$ this interaction (S3) acquires the usual form $H_{\mathrm{so}} = \beta(\sigma_x k_x - \sigma_y k_y)$, and in the vicinity of, e.g., the $(\pi/a, 0)$ point one obtains $H_{\mathrm{so}} = -\beta[\sigma_x(k_x - \pi/a) + \sigma_y k_y]$.

The Hamiltonian of anisotropic tight-binding model, which includes DSOI and an in-plane magnetic field B parallel to the x-axis can be written as

$$H = \varepsilon_{\mathbf{k}}^{(0)} + \frac{\beta}{a}\left[\sigma_x \sin(k_x a) - \sigma_y \sin(k_y a)\right] + \lambda B \sigma_x, \tag{S4}$$

with λ being the corresponding $g-$factor. The energy of electrons with the Hamiltonian (S4) becomes

$$\varepsilon_{\pm}(\mathbf{k}) = -w\cos(k_x a) - t\cos(k_y a) \pm \sqrt{\frac{\beta^2}{a^2}\left[\sin^2(k_x a) + \sin^2(k_y a)\right] + \frac{2\beta}{a}\lambda B \sin(k_x a) + \lambda^2 B^2}, \tag{S5}$$

where the $\pm$ signs correspond to different energy subbands. The electron energy spectrum in k_x and k_y directions is shown in Fig. S7.

The spin-orbit interaction is much more pronounced in the x-direction due to a larger effective mass resulting in a stronger extension along this axis. Obviously, the joint effect of spin-orbit coupling and magnetic field breaks the $k_x \to -k_x$ symmetry, which can be important for the superconductivity usually related to the pairing of electrons in the $\mathbf{k}$ and $-\mathbf{k}$ states.

The electron spin-dependent Fermi surfaces for the Fermi energy μ are determined by the $\varepsilon_{\mathbf{k},\pm} = \mu$ line with the density of states (DOS) in "+" and "-" spin-split subbands given by

$$\rho_{\pm}(\varepsilon) = \int \frac{d^2 k}{(2\pi)^2}\, \delta\left[\varepsilon - \varepsilon_{\pm}(\mathbf{k})\right]. \tag{S6}$$

Since the density of states exhibits a van Hove singularities at the saddle points of $\varepsilon_{\pm}(\mathbf{k})$, the superconducting transition temperature can be expected to be strongly sensitive to the positions of these singularities.

Energy spectrum near the van Hove singularities

In the following consideration, we focus mainly on the vicinity of the $\mathbf{k} = (\pm\pi/a, 0)$−point, where the van Hove singularities are located. Therefore, we present the energy spectrum and the Fermi surface centered at the $k_x = -\pi/a$ point of the Brillouin zone. Figure S8 shows how these characteristics of the bands change with the variation of the magnetic field for the $\varepsilon_+(\mathbf{k})$ subband.

The energy maxima in the left panel of Fig. S8 are the saddle points of the function $\varepsilon_+(\mathbf{k})$, corresponding to the singularities in the DOS shown in the left panel of Fig. S9. We see that the magnetic field deforms the Fermi surface but does not break the $k_y \to -k_y$ symmetry, $\varepsilon_+(k_y) = \varepsilon_+(-k_y)$. Figure S9 (right panel) presents the B−dependence of DOS at the Fermi level for a constant μ.

Apparently, the magnetic field can strongly enhance $\rho_+(\mu)$, which can cause an increase of the superconducting transition temperature with the applied magnetic field.

Cooper pairing and the critical temperature for in-plane magnetic field

Due to the lack of inversion symmetry with $\varepsilon_{\pm}(\mathbf{k}) \neq \varepsilon_{\pm}(-\mathbf{k})$ for $B \neq 0$ [cf. Eq. (S5)], the in-plane magnetic field precludes Cooper pairing of electrons with $\mathbf{k}$ and $-\mathbf{k}$ wavevectors but allows coupling of the states with nonzero total momentum. However, if the field B is relatively weak, it is still possible that electrons in $(\mathbf{k} + \mathbf{q})$ and $(-\mathbf{k} + \mathbf{q})$ states can be coupled, where $\mathbf{q}$ is a $\mathbf{k}$-dependent vector characterizing the Cooper pair with nonzero momentum. Thus, we begin with an analysis of the finite-momentum pairing and then consider how it occurs in the presence of an external magnetic field and spin-orbit coupling.

To obtain the corresponding transition temperature, we consider a renormalization of the electron-electron interaction in the Cooper channel. Following Ref. (*40*), this renormalization can be presented as (here g_0 and g denote the bare and the renormalized coupling constants,

respectively):

$$g = g_0 - g_0 g k_B T \sum_n \int \frac{d^2k}{(2\pi)^2} G(i\varepsilon_n, \mathbf{q}+\mathbf{k})\, G(-i\varepsilon_n, \mathbf{q}-\mathbf{k}), \tag{S7}$$

where k_B is the Boltzmann constant and

$$G(i\varepsilon_n, \mathbf{k}) = (i\varepsilon_n - \xi_{\mathbf{k}})^{-1} \tag{S8}$$

is the Matsubara Green's function with $\varepsilon_n = (2n+1)k_BT$ and $\xi_{\mathbf{k}}$ being the electron energy measured from the chemical potential, $\xi_{\mathbf{k}} = \varepsilon_{\mathbf{k}} - \mu$.

The solution of (S7) is

$$g = \frac{g_0}{1 + g_0 I}, \tag{S9}$$

where

$$I = k_B T \sum_n \int \frac{d^2k}{(2\pi)^2} G(i\varepsilon_n, \mathbf{q}+\mathbf{k})\, G(-i\varepsilon_n, \mathbf{q}-\mathbf{k}). \tag{S10}$$

The pole of the function in Eq. (S9), where $g_0 I + 1 = 0$, determines the critical temperature of the superconducting transition. Next, in Eq. (S10) we use the formula of transition from the sum over Matsubara frequencies ε_n to the integration in the complex ε-plane

$$\sum_n \phi(i\varepsilon_n) = \frac{i}{k_BT} \int_C f(\varepsilon)\, \phi(\varepsilon)\, \frac{d\varepsilon}{2\pi}, \tag{S11}$$

where $f(\varepsilon) = (e^{\varepsilon/k_BT} + 1)^{-1}$ is the Fermi-Dirac function, and C is the corresponding contour (see Ref. (*40*) for details). This contour can be transformed into the contour C' around the poles of the Matsubara Green's functions in Eq. (S10). Then we obtain

$$I = -i \sum_{\mathbf{k}} \int_{C'} \frac{f(\varepsilon)}{(\varepsilon - \xi_{\mathbf{q}+\mathbf{k}})(\varepsilon + \xi_{\mathbf{q}-\mathbf{k}})} \frac{d\varepsilon}{2\pi}, \tag{S12}$$

and integration over C' yields:

$$I = -\sum_{\mathbf{k}'} \frac{f(\xi_{\mathbf{q}+\mathbf{k}}) - f(-\xi_{\mathbf{q}-\mathbf{k}})}{\xi_{\mathbf{q}+\mathbf{k}} + \xi_{\mathbf{q}-\mathbf{k}}}. \tag{S13}$$

Equations (S11) and (S13) lead to the equation for critical temperature T_c as:

$$g_0 \sum_{\mathbf{k}'} \frac{f(\xi_{\mathbf{q}+\mathbf{k}}) - f(-\xi_{\mathbf{q}-\mathbf{k}})}{\xi_{\mathbf{q}+\mathbf{k}} + \xi_{\mathbf{q}-\mathbf{k}}} = 1. \tag{S14}$$

For the pairing of states with equal energies, where $\xi_{\mathbf{q}+\mathbf{k}} = \xi_{\mathbf{q}-\mathbf{k}}$, using the identity

$$f(E) - f(-E) = -\tanh \frac{E}{2k_B T} \tag{S15}$$

we obtain

$$I = \sum_{\mathbf{k}} \frac{\tanh(\xi_{\mathbf{q}+\mathbf{k}}/2k_B T)}{2\xi_{\mathbf{q}+\mathbf{k}}}. \tag{S16}$$

Correspondingly, at $\mathbf{q} = \mathbf{0}$, the equation for T_c acquires the usual BSC form

$$-g_0 \sum_{\mathbf{k}} \frac{\tanh(\xi_{\mathbf{k}}/2k_B T_c)}{2\xi_{\mathbf{k}}} = 1, \tag{S17}$$

which has solution only for $g_0 < 0$.

Next, we consider the above approach for the model of pairing corresponding to the electron spectrum in Eq. (S5) taking into account that this spectrum consists of two spin-related subbands described by the transition temperatures $T_c^{\pm}$, correspondingly, as determined by:

$$g_0 \sum_{\mathbf{k}} \frac{f(\xi_{\mathbf{k}+\mathbf{q},\pm}) - f(-\xi_{-\mathbf{k}+\mathbf{q},\pm})}{\xi_{\mathbf{k}+\mathbf{q},\pm} + \xi_{-\mathbf{k}+\mathbf{q},\pm}} = 1, \tag{S18}$$

where $f(\varepsilon) = (e^{\varepsilon/k_B T_c^{\pm}} + 1)^{-1}$ is taken at the corresponding transition temperature. Using the identity (S15) we can rewrite Eq. (S18) into the form

$$-g_0 \sum_{\mathbf{k}} \frac{1}{2\xi_{\mathbf{k}+\mathbf{q},\pm}} \tanh \frac{\xi_{\mathbf{k}+\mathbf{q},\pm}}{2k_B T_c^{\pm}} = 1. \tag{S19}$$

We specify the vector $\mathbf{q}_{\pm} = (q_{x,\pm}, q_{y,\pm})$ by the same-energy condition

$$\xi_{\mathbf{k}+\mathbf{q}_{\pm},\pm} = \xi_{-\mathbf{k}+\mathbf{q}_{\pm},\pm}, \tag{S20}$$

corresponding to the pairing of the states with the same energy assuming in addition that they have opposite spins, and by the requirement $q_{y,\pm} = 0$ since a weak nonzero magnetic field shifts the Fermi surface in the x-direction. From (S20), using Eq. (S5) and assuming $\lambda B \ll \beta/a$, we obtain

$$q_{x,\pm} = \mp \frac{\beta\lambda B}{a^2\eta_{\mathbf{k}}(w \pm \beta^2 \cos(k_y a)/a^2\eta_{\mathbf{k}})}, \tag{S21}$$

where we denoted

$$\eta_{\mathbf{k}} = \sqrt{\frac{\beta^2}{a^2}\left[\sin^2(k_x a) + \sin^2(k_y a)\right] + \lambda^2 B^2}. \tag{S22}$$

The density of states in the upper subband $\rho_+(\varepsilon)$ is larger than that for the lower subband since the corresponding Fermi surface is close to the k_x-axis. As a result, it produces a higher critical temperature $T_c^+ > T_c^-$, and therefore we concentrate on the upper energy subband and omit the corresponding subband index. The results of the numerical calculation of T_c^+ using Eq. (S14) are presented in Fig. S10 for $q_{x,+}$ from Eq. (S21).

We conclude that in some energy intervals, depending on the choice of parameters, the dependence of T_c on the magnetic field is very unusual: it decreases at weak B, then goes up and drops again with the increasing field. This initial decrease and the following increase in T_c can be attributed to the interplay of a decreasing effective electron-electron interaction and an increasing density of states at the Fermi level due to the van Hove singularity. The peak of each curve in Fig. S10 corresponds to the field at which the Fermi level intersects the van Hove singularity.

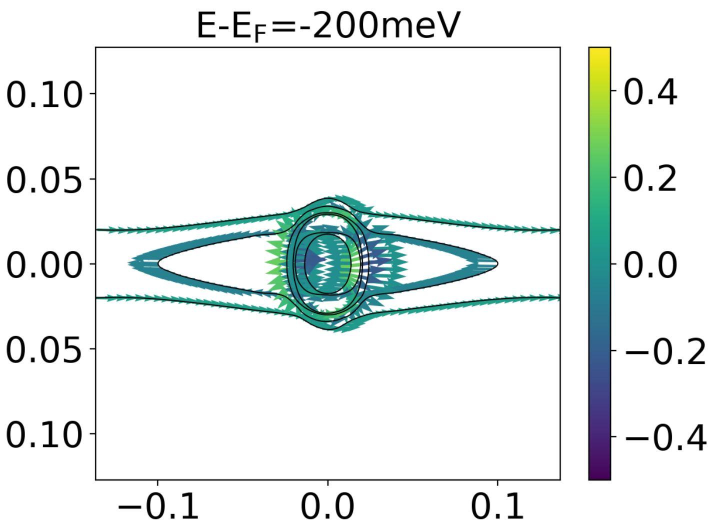

Fig. S1: Fermi surface cross section of $(LTiO)_1/(KTO)_1$ (110) and its spin textures $S_i(\mathbf{k})$ calculated from first principles within the $k_z = 0$ plane. The color indicates the out-of-plane component $S_z(\mathbf{k})$.

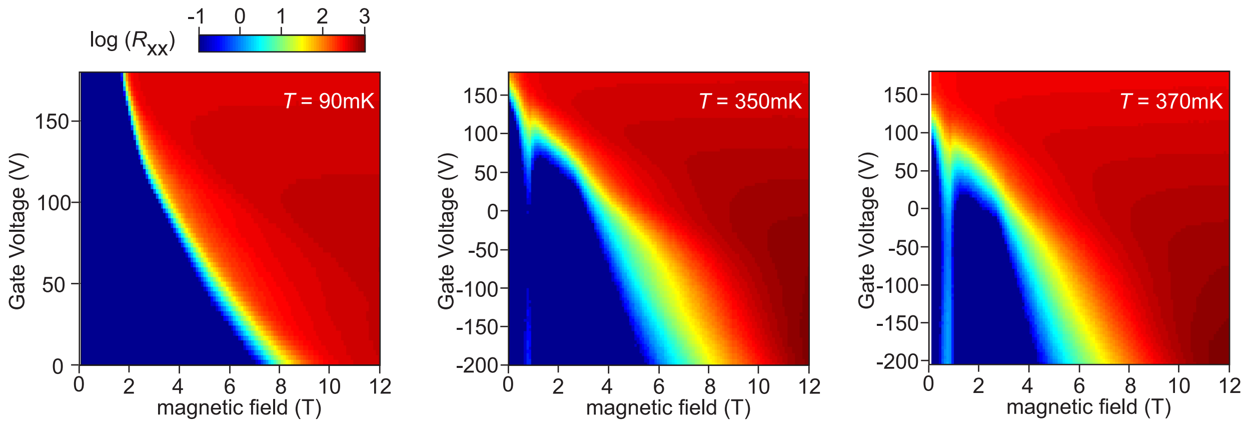

Fig. S2: Reentrant response for current along [001].

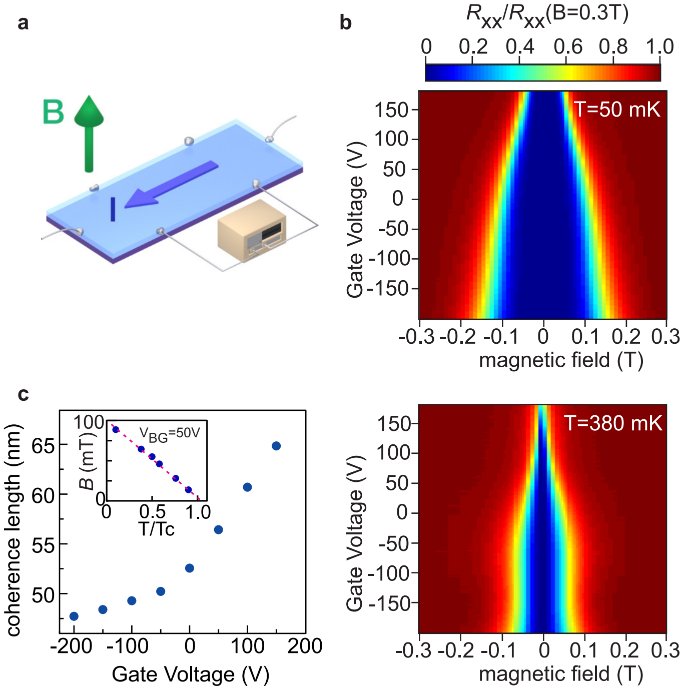

Fig. S3: Determination of effective coherence length.

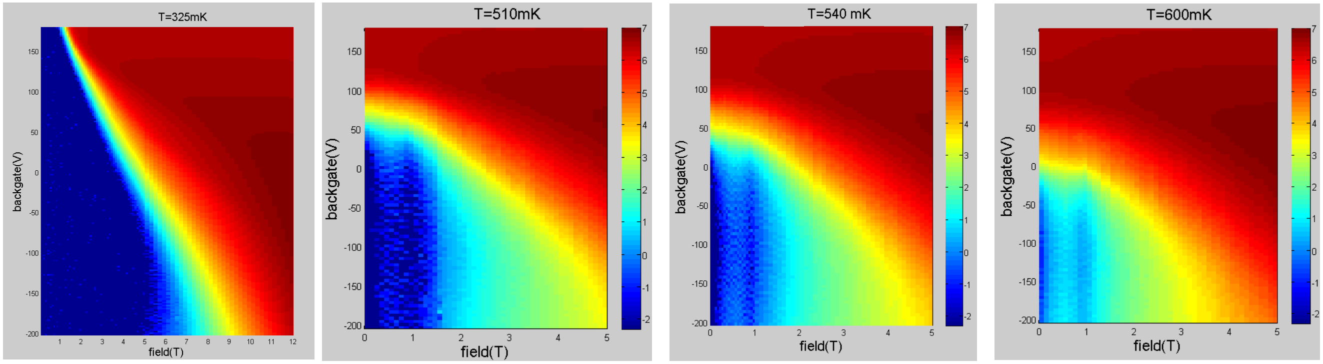

Fig. S4: Reproducible re-entrant state.

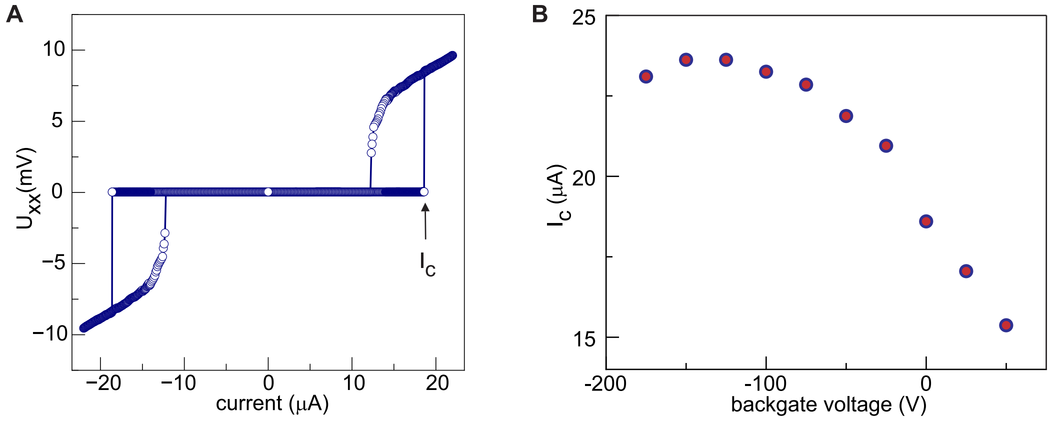

Fig. S5: Current-voltage characteristics of a superconducting state at $B = 0$ T and $T = 90$ mK.

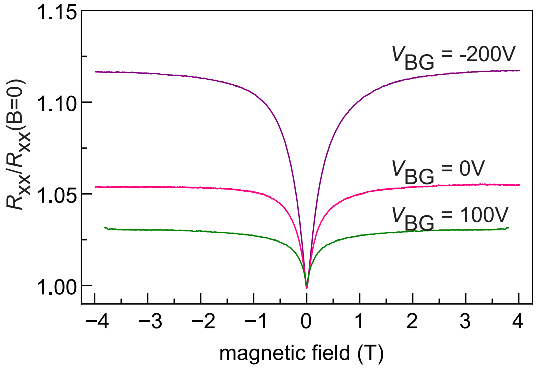

Fig. S6: Magnetoresistance measured at $T \approx 800$ mK with magnetic field applied perpendicularto the interface.

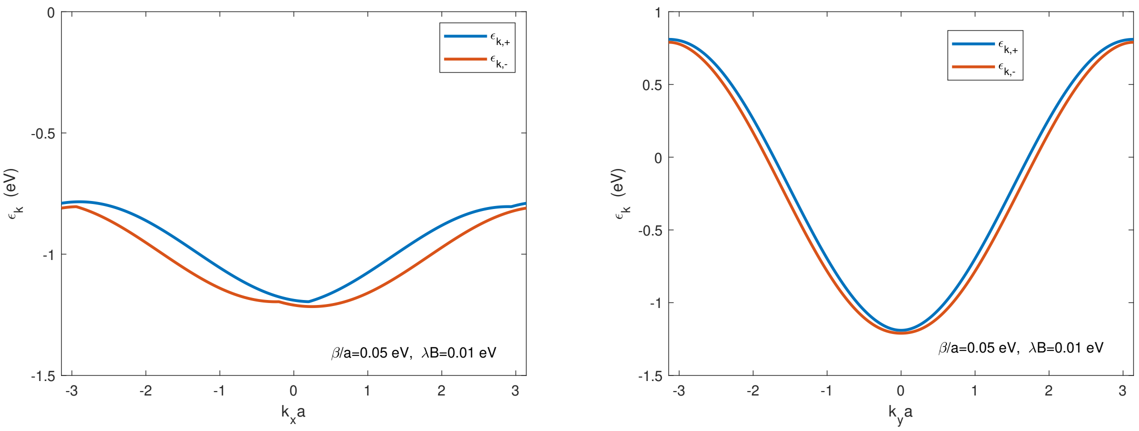

Fig. S7: Electron spectrum $\varepsilon_{\pm}(k_x)$ for $k_y = 0$ (left panels) and $\varepsilon_{\pm}(k_y)$ for $k_x = 0$ (right panels). Spin splitting is due to both DSOI and magnetic field. The band parameters are $t = 1$ eV and $w = 0.2$ eV.

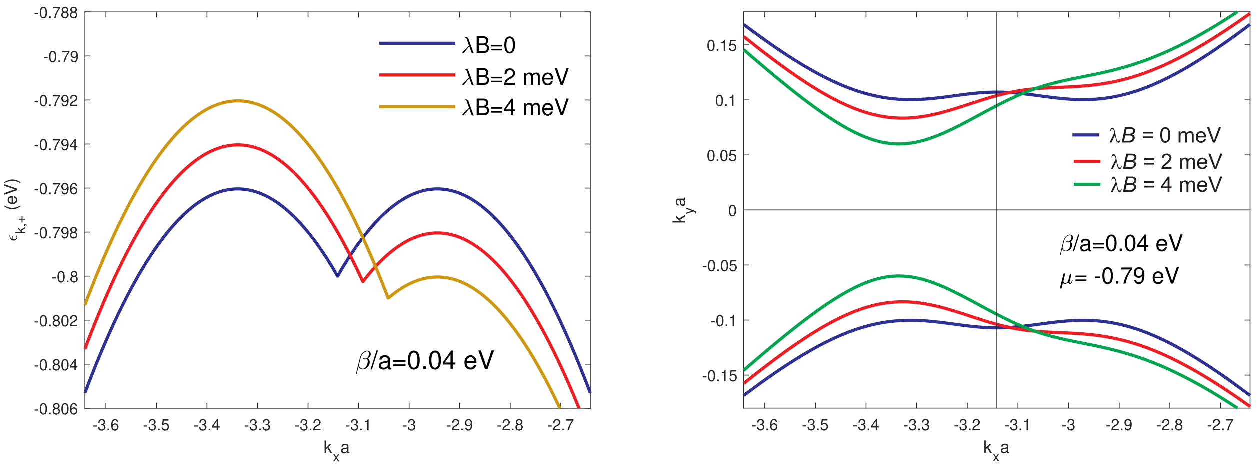

Fig. S8: Electron energy spectrum $\varepsilon_{+}(k_x)$ for $k_y = 0$ (left panel) and the variation of the Fermi surface with magnetic field [$\lambda B = 0$ (blue), 2 meV (red), 4 meV (green)] (right panel). The DSOI parameter $\beta/a = 0.04$ eV.

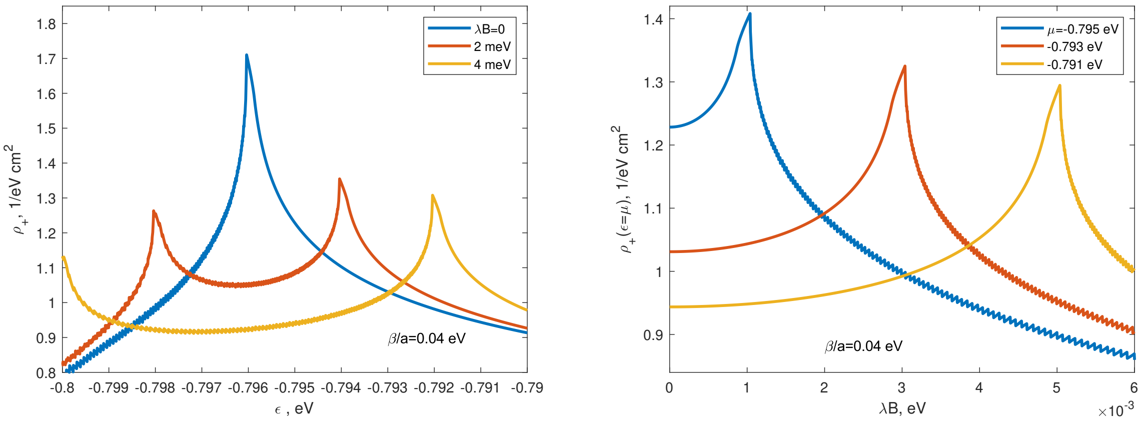

Fig. S9: Variation of the density of states $\rho_+(\varepsilon)$ with magnetic field (left panel) and the density of states at the Fermi level $\rho_+(\varepsilon = \mu)$ as a function of magnetic field (right panel).

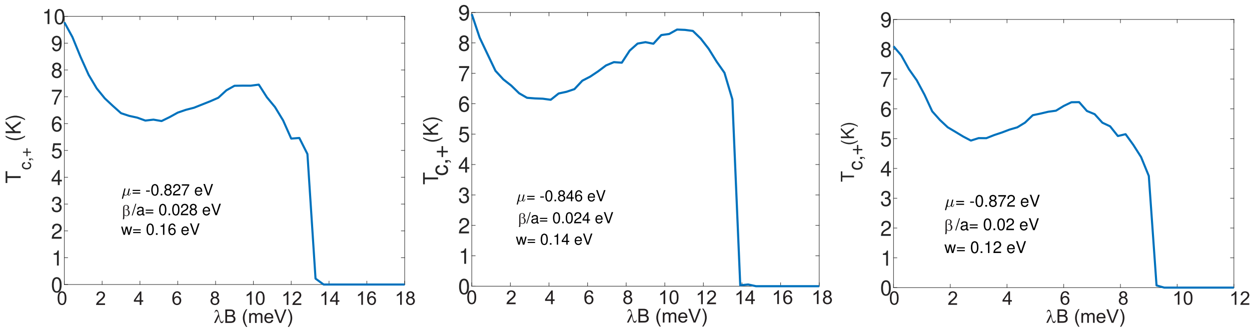

Fig. S10: The dependence of T_c on magnetic field for different choice of parameters.

REFERENCES

1. H. W. Meul, C. Rossel, M. Decroux, Ø. Fischer, G. Remenyi, A. Briggs, Observation of magnetic-field-induced superconductivity. *Phys. Rev. Lett.* **53**, 497–500 (1984).

2. T. Konoike, S. Uji, T. Terashima, M. Nishimura, S. Yasuzuka, K. Enomoto, H. Fujiwara, B. Zhang, H. Kobayashi, Magnetic-field-induced superconductivity in the antiferromagnetic organic superconductor κ-$(BETS)_2FeBr_4$. *Phys. Rev. B* **70**, 094514 (2004).

3. F. Lévy, I. Sheikin, B. Grenier, A. D. Huxley, Magnetic field-induced superconductivity in the ferromagnet URhGe. *Science* **309**, 1343–1346 (2005).

4. S. Ran, I.-L. Liu, Y. S. Eo, D. J. Campbell, P. M. Neves, W. T. Fuhrman, S. R. Saha, C. Eckberg, H. Kim, D. Graf, F. Balakirev, J. Singleton, J. Paglione, N. P. Butch, Extreme magnetic field-boosted superconductivity. *Nat. Phys.* **15**, 1250–1254 (2019).

5. V. Jaccarino, M. Peter, Ultra-high-field superconductivity. *Phys. Rev. Lett.* **9**, 290–292 (1962).

6. J. J. Sanchez, G. Fabbris, Y. Choi, J. M. DeStefano, E. Rosenberg, Y. Shi, P. Malinowski, Y. Huang, I. I. Mazin, J.-W. Kim, J.-H. Chu, P. J. Ryan, Strain-switchable field-induced superconductivity. *Sci. Adv.* **9**, eadj5200 (2023).

7. S. Khim, J. F. Landaeta, J. Banda, N. Bannor, M. Brando, P. M. R. Brydon, D. Hafner, R. Küchler, R. Cardoso-Gil, U. Stockert, A. P. Mackenzie, D. F. Agterberg, C. Geibel, E. Hassinger, Field-induced transition within the superconducting state of $CeRh_2As_2$. *Science* **373**, 1012–1016 (2021).

8. S. Uji, H. Shinagawa, T. Terashima, T. Yakabe, Y. Terai, M. Tokumoto, A. Kobayashi, H. Tanaka, H. Kobayashi, Magnetic-field-induced superconductivity in a two-dimensional organic conductor. *Nature* **410**, 908–910 (2001).

9. T. Helm, M. Kimata, K. Sudo, A. Miyata, J. Stirnat, T. Förster, J. Hornung, M. König, I. Sheikin, A. Pourret, G. Lapertot, D. Aoki, G. Knebel, J. Wosnitza, J.-P. Brison, Field-induced compensation of magnetic exchange as the possible origin of reentrant superconductivity in UTe_2. *Nat. Commun.* **15**, 37 (2024).

10. L. P. Gor'kov, E. I. Rashba, Superconducting 2D system with lifted spin degeneracy: Mixed singlet-triplet state. *Phys. Rev. Lett.* **87**, 037004 (2001).

11. J. M. Lu, O. Zheliuk, I. Leermakers, N. F. Q. Yuan, U. Zeitler, K. T. Law, J. T. Ye, Evidence for two-dimensional Ising superconductivity in gated MoS_2. *Science* **350**, 1353–1357 (2015).

12. X. Xi, Z. Wang, W. Zhao, J.-H. Park, K. T. Law, H. Berger, L. Forró, J. Shan, K. F. Mak, Ising pairing in superconducting $NbSe_2$ atomic layers. *Nat. Phys.* **12**, 139–143 (2016).

13. Y. Saito, Y. Nakamura, M. S. Bahramy, Y. Kohama, J. Ye, Y. Kasahara, Y. Nakagawa, M. Onga, M. Tokunaga, T. Nojima, Y. Yanase, Y. Iwasa, Superconductivity protected by spin–valley locking in ion-gated MoS_2. *Nat. Phys.* **12**, 144–149 (2016).

14. M. Smidman, M. B. Salamon, H. Q. Yuan, D. F. Agterberg, Superconductivity and spin–orbit coupling in non-centrosymmetric materials: A review. *Rep. Prog. Phys.* **80**, 036501 (2017).

15. Y. Saito, T. Nojima, Y. Iwasa, Highly crystalline 2D superconductors. *Nat. Rev. Mater.* **2**, 16094 (2017).

16. Y. Cao, V. Fatemi, S. Fang, K. Watanabe, T. Taniguchi, E. Kaxiras, P. Jarillo-Herrero, Unconventional superconductivity in magic-angle graphene superlattices. *Nature* **556**, 43–50 (2018).

17. P. Wan, O. Zheliuk, N. F. Q. Yuan, X. Peng, L. Zhang, M. Liang, U. Zeitler, S. Wiedmann, N. E. Hussey, T. T. M. Palstra, J. Ye, Orbital Fulde–Ferrell–Larkin–Ovchinnikov state in an Ising superconductor. *Nature* **619**, 46–51 (2023).

18. Y. Cao, J. M. Park, K. Watanabe, T. Taniguchi, P. Jarillo-Herrero, Pauli-limit violation and re-entrant superconductivity in moiré graphene. *Nature* **595**, 526–531 (2021).

19. C. Liu, X. Yan, D. Jin, Y. Ma, H.-W. Hsiao, Y. Lin, T. M. Bretz-Sullivan, X. Zhou, J. Pearson, B. Fisher, J. S. Jiang, W. Han, J.-M. Zuo, J. Wen, D. D. Fong, J. Sun, H. Zhou, A. Bhattacharya, Two-dimensional superconductivity and anisotropic transport at $KTAO_3$(111) interfaces. *Science* **371**, 716–721 (2021).

20. C. Liu, X. Zhou, D. Hong, B. Fisher, H. Zheng, J. Pearson, J. S. Jiang, D. Jin, M. R. Norman, A. Bhattacharya, Tunable superconductivity and its origin at $KTAO_3$ interfaces. *Nat. Commun.* **14**, 951 (2023).

21. Z. Chen, Z. Liu, Y. Sun, X. Chen, Y. Liu, H. Zhang, H. Li, M. Zhang, S. Hong, T. Ren, C. Zhang, H. Tian, Y. Zhou, J. Sun, Y. Xie, Two-dimensional superconductivity at the $LaA1O_3$/$KTAO_3$(110) heterointerface. *Phys. Rev. Lett.* **126**, 026802 (2021).

22. G. Zhang, L. Wang, J. Wang, G. Li, G. Huang, G. Yang, H. Xue, Z. Ning, Y. Wu, J.-P. Xu, Y. Song, Z. An, C. Zheng, J. Shen, J. Li, Y. Chen, W. Li, Spontaneous rotational symmetry breaking in $KTaO_3$ heterointerface superconductors. *Nat. Commun.* **14**, 3046 (2023).

23. X. Hua, Z. Zeng, F. Meng, H. Yao, Z. Huang, X. Long, Z. Li, Y. Wang, Z. Wang, T. Wu, Z. Weng, Y. Wang, Z. Liu, Z. Xiang, X. Chen, Superconducting stripes induced by ferromagnetic proximity in an oxide heterostructure. *Nat. Phys.* **20**, 957–963 (2024).

24. D. Maryenko, I. V. Maznichenko, S. Ostanin, M. Kawamura, K. S. Takahashi, M. Nakamura, V. K. Dugaev, E. Y. Sherman, A. Ernst, M. Kawasaki, Superconductivity at epitaxial $LaTiO_3$–$KTaO_3$ interfaces. *APL Mater.* **11**, 061102 (2023).

25. A. M. Clogston, Upper limit for the critical field in hard superconductors. *Phys. Rev. Lett.* **9**, 266–267 (1962).

26. B. S. Chandrasekhar, A note on the maximum critical field of high-field superconductors. *Appl. Phys. Lett.* **1**, 7–8 (1962).

27. L. Li, C. Richter, J. Mannhart, R. C. Ashoori, Coexistence of magnetic order and two-dimensional superconductivity at $LaAlO_3$/$SrTiO_3$ interfaces. *Nat. Phys.* **7**, 762–766 (2011).

28. J. A. Bert, B. Kalisky, C. Bell, M. Kim, Y. Hikita, H. Y. Hwang, K. A. Moler, Direct imaging of the coexistence of ferromagnetism and superconductivity at the $LaAlO_3$/$SrTiO_3$ interface. *Nat. Phys.* **7**, 767–771 (2011).

29. O. Krebs, P. Voisin, Giant optical anisotropy of semiconductor heterostructures with no common atom and the quantum-confined pockels effect. *Phys. Rev. Lett.* **77**, 1829–1832 (1996).

30. U. Rössler, J. Kainz, Microscopic interface asymmetry and spin-splitting of electron subbands in semiconductor quantum structures. *Solid State Commun.* **121**, 313–316 (2002).

31. L. E. Golub, E. L. Ivchenko, Spin splitting in symmetrical SiGe quantum wells. *Phys. Rev. B* **69**, 115333 (2004).

32. T. Salamone, H. G. Hugdal, S. H. Jacobsen, M. Amundsen, High magnetic field superconductivity in a two-band superconductor. *Phys. Rev. B* **107**, 174516 (2023).

33. J. Clepkens, H.-Y. Kee, Finite-momentum and field-induced pairings in orbital-singlet spin-triplet superconductors. *Phys. Rev. B* **109**, 214512 (2024).

34. A. A. Vargas-Paredes, A. A. Shanenko, A. Vagov, M. V. Milošević, A. Perali, Crossband versus intraband pairing in superconductors: Signatures and consequences of the interplay. *Phys. Rev. B* **101**, 094516 (2020).

35. G. Kresse, J. Furthmüller, Efficient iterative schemes for ab initio total-energy calculations using a plane-wave basis set. *Phys. Rev. B* **54**, 11169–11186 (1996).

36. J. P. Perdew, K. Burke, M. Ernzerhof, Generalized gradient approximation made simple. *Phys. Rev. Lett.* **77**, 3865–3868 (1996).

37. D. Hobbs, G. Kresse, J. Hafner, Fully unconstrained noncollinear magnetism within the projector augmented-wave method. *Phys. Rev. B* **62**, 11556–11570 (2000).

38. I. V. Maznichenko, S. Ostanin, D. Maryenko, V. K. Dugaev, E. Y. Sherman, P. Buczek, I. Mertig, M. Kawasaki, A. Ernst, Emerging two-dimensional conductivity at the interface between mott and band insulators. *Phys. Rev. Lett.* **132**, 216201 (2024).

39. I. V. Maznichenko, A. Ernst, D. Maryenko, V. K. Dugaev, E. Y. Sherman, P. Buczek, S. S. P. Parkin, S. Ostanin, Fragile altermagnetism and orbital disorder in Mott insulator $LaTiO_3$. *Phys. Rev. Mater.* **8**, 064403 (2024).

40. A. A. Abrikosov, L. P. Gor'kov, I. E. Dzyaloshinski. *Methods of Quantum Field Theory in Statistical Physics* (Dover, 1963).
